# Parent to Offspring Fear Transmission via Modeling in Early Life: A Systematic Review and Meta-Analysis

**DOI:** 10.1007/s10567-023-00448-1

**Published:** 2023-07-27

**Authors:** Cosima Anna Nimphy, Marianna Venetikidi, Bernet Elzinga, Willem van der Does, Evin Aktar

**Affiliations:** 1 Department of Clinical Psychology, Leiden University, Leiden, The Netherlands; 2 Leiden Institute for Brain and Cognition (LIBC), Leiden University, Leiden, The Netherlands; 3 Leiden University Treatment Center (LUBEC), Leiden University, Leiden, The Netherlands

**Keywords:** Modeling, Vicarious Learning, Fear, Infant, Temperament, Parental Anxiety

## Abstract

Infants can acquire fears vicariously by observing parents’ fearful reactions to novel stimuli in everyday situations (i.e., modeling). To date, no systematic or meta-analytic review examined the role of modeling in parent–child transmission of fear and avoidance in early life. In our systematic review and meta-analysis, we aimed to investigate the effect of modeling parents’ fearful reactions on infants’ acquisition of fear and avoidance of novel stimuli and explore the moderation of this effect by child behavioral inhibition (BI) and parent trait anxiety. The search conducted in Web Of Science, Pubmed, Embase, and PsycINFO revealed 23 eligible studies for the systematic review and 19 for the meta-analysis. Eligible studies included published studies that measured infant fear and avoidance (infants aged up to 30 months) of novel stimuli following exposure to parental fearful expressions. Meta-analysis findings revealed a significant causal effect of modeling of parental fear on infants’ fear [*g* = .44] and avoidance of novel stimuli [*g* = .44]. The findings support moderation by child BI on infant avoidance (not fear) acquisition, with the effects being larger for infants with higher BI. However, this moderation was only found, when including both experimental and correlational studies *(p* > .05*)*, but not when exclusively including experimental studies *(p* = .17*)*. This meta-analysis provides support for early parent-to-offspring fear transmission: a causal small to medium effect of parents’ fearful reactions was shown on infants’ fear and avoidance of novel stimuli. Elucidating parent-to-offspring anxiety transmission pathways can inform us about potential fear reduction and prevention strategies.

**Supplementary Information:**

The online version contains supplementary material available at 10.1007/s10567-023-00448-1.

## Introduction

Anxiety disorders run in families (Eley et al., 2015; Gregory & Eley, 2011)*.* Children of parents with a current or lifetime anxiety disorder are up to three times more likely to develop an anxiety disorder than children of non-anxious parents (Lawrence et al., 2019; Telman et al., 2018). Studies on familial aggregation of anxiety have investigated the role of both genetic and environmental influences (Eley et al., 2015; Gregory & Eley, 2011; Hettema et al., 2001). Genetic transmission contributes significantly to family aggregation of anxiety (Hettema et al., 2001; Vasey & Dadds, 2001). Twin studies suggest heritability estimates between approximately 30 and 50% (Shimada-Sugimoto et al., 2015), which can vary depending on sex, age, and how anxiety was assessed (Gregory & Eley, 2011). Twin studies also stress the importance of shared environment in family aggregation of anxiety (Gregory & Eley, 2011). Recently, a novel children-of-twins study investigated the relative influences of genetic and environmental factors in the anxiety transmission from parent to child (Eley et al., 2015). They reported that environmental factors mainly account for the parent–child transmission of anxiety and should be focused on in subsequent research.

Environmental mechanisms can be conceptualized within the broader context of fear acquisition frameworks and social learning theory (Bandura & Walters, 1977; Olsson et al., 2007; Rachman, 1977). In addition to their first-hand aversive experiences with novel stimuli, children can acquire fears indirectly via others, particularly parents (so-called social fear learning, Olsson et al., 2007; Rachman, 1977). Social fear learning involves verbal communications signaling threat (i.e., parent saying ‘this is scary, right?’) (Muris & Field, 2010), as well as modeling of fear expressions (also referred to as vicarious learning or observational learning, see Askew & Field, 2008). Research focusing on parent–child transmission of fear via environmental mechanisms has most frequently investigated parental modeling of fearful/avoidant expressions and behavior as a fear learning pathway (Fisak & Grills-Taquechel, 2007). In our meta-analysis, we investigated the effect of modeling parents’ fearful reactions on infants’ acquisition of fear and avoidance of novel stimuli.

Parent to offspring fear transmission via modeling might be especially relevant in children’s first years of life for several reasons. First, the first two years have been highlighted as a sensitive/vulnerable period for exposure to parental fearful and anxious expressions and behavior (Aktar & Bögels, 2017). At this age, infants’ rapid and experience-driven development of emotional brain systems, as well as enhanced face processing of caregivers might make infants particularly vulnerable to parental anxious signals (Leppänen, 2011; Leppänen & Nelson, 2009). Second, the emergence of social referencing abilities in infants between approximately 10–14 months may be particularly relevant to the development of fear, as infants actively seek out information from parents when confronted with novelty/ambiguity (Fisak & Grills-Taquechel, 2007). Third, while infants may already understand some parental verbal cues before and in the early phases of language acquisition, parent–child communication largely depends on facial expressions, followed by gestures and body language (Feinman et al., 1992). As mentioned by Rachman (1977), with the development of language abilities, social fear learning via verbal threat information becomes a relevant fear learning pathway. By focusing on the first years of life, we can quantify the effect of vicarious learning in early life while minimizing the influence of parental verbal threat information (which would vary greatly across age). While previous literature discussed the causal role of vicarious learning in fear acquisition of infants (Aktar & Bögels, 2017; Debiec & Olsson, 2017; Fisak & Grills-Taquechel, 2007; LoBue, et al., 2019; Murray et al., 2009), this effect has not been systematically assessed or quantified for this age range.

Infant modeling of parents’ non-verbal fear expressions has been studied in so-called social referencing paradigms (Gerull & Rapee, 2002; Murray et al., 2008). In this paradigm, infants are directly exposed to parents’ fearful response to a novel stimulus (i.e., ambiguous toy or stranger) and then themselves exposed to the novel stimulus. Multiple behavioral components of infant responses to these stimuli are measured, such as infants’ affective response (facial, bodily, or vocal expressions of fear, i.e., crying), as well as their avoidant response (facial or bodily avoidance of stimulus, i.e., turning or moving away from stimulus). The avoidant reaction to a novel stimulus can be seen as a regulatory response to reduce distress and escape the stimulus (Aktar & Pérez-Edgar, 2020; Klinnert, 1984). From here onward, based on previous studies, we will refer to the affective component as “fear”, whereas the regulatory avoidant response will be referred to as “avoidance” (Aktar et al., 2013; De Rosnay et al., 2006; Murray et al., 2008). Parental expressions of fear toward a novel stimulus might not necessarily increase both infant fear and avoidance toward the stimulus at the same time (Walden & Ogan, 1988), meaning infant fear and avoidance do not have to co-occur. Therefore, we decided to investigate the effect of parents’ fearful reactions to novel stimuli on infants’ acquisition of fear and avoidance of these stimuli separately.

Two lines of research have addressed fear transmission in infancy via vicarious learning. The first line of studies investigates typically developing infants and uses experimental designs where parental emotional displays toward novel/ambiguous stimuli are manipulated (via training) (Dubi et al., 2008; Egliston & Rapee, 2007; Feinman & Lewis, 1983; Fisak & Grills-Taquechel, 2007; Gerull & Rapee, 2002). By randomly assigning parents to manipulation and control condition, the experimental studies control for factors such as the genetic transmission and the learning history of parental behaviors affecting the child. The second line of studies uses naturalistic observations in clinical samples of anxious parents with infants, during novel/ambiguous situations, instead of manipulating/training parental expressions (Aktar et al., 2013, 2014; de Rosnay et al., 2006; Murray et al., 2008). In the correlational studies, we might get a more representative insight on the impact parents’ natural fear responses to novel stimuli have on infants fear and avoidance. The two lines are complementary as the first one allows causal inferences and the second aims to capture the transmission of anxiety in real life. Consequently, we will examine both lines of research in a systematic review and meta-analysis.

It is important to note that parent-to-child transmission of fear is inherently an evolutionary-adaptive mechanism that helps the infant to become aware of and stay away from dangerous situations, maximizing survival (Feinman, 1985). However, if parents have an anxiety disorder, which is typically characterized by excessive fear and overestimation of danger (APA, 2015), they might expose their children to anxiety signals in the absence of actual danger. A previous study found that parents with social phobia were more likely to display threat signals when exposed to strangers interacting with their infant in an approach task, than parents without social phobia (Murray, et al., 2008). Infants may acquire fear of ambiguous situations as a result of repeatedly observing parents’ anxiety signals (Aktar & Bögels, 2017; Murray et al., 2009). Furthermore, over time infants of anxious parents might learn to pay attention to threat over safety signals or interpret the signals more negatively (Aktar, 2022; Creswell et al., 2010). If the child’s fear is not in proportion to the severity of the threat, persists, and interferes with daily functioning, this fear response can be regarded as maladaptive (Kiel & Kalomiris, 2019). Therefore, the effect of parental modeling of fear/anxiety on child acquisition to novel stimuli might be stronger for infants of anxious parents than of non-anxious parents.

Infants are not only passive receivers of parents’ fear and anxiety signals but their characteristics play a role in the intergenerational transmission of fear too (Reynolds et al., 2018). Behavioral inhibition (BI) is the strongest temperamental predictor of the later development of social anxiety (Clauss & Blackford, 2012). BI is defined as a fearful and avoidant style of reacting to ambiguous stimuli (Fox et al., 2005). Theoretical models indicate that infants with BI would be more susceptible to environmental stressors, including parental anxiety signals (Belsky & Pluess, 2009; Ingram & Luxton, 2005; Nigg, 2006). Furthermore, infants’ fearful temperament is consistently found to strengthen the impact of parents’ anxious expressions on infants’ vicarious acquisition of anxiety (Aktar et al., 2013; De Rosnay et al., 2006; Möller et al., 2014).

Although the current focus is on modeling, it is only one of the many environmental mechanisms that may alone or in interaction contribute to parent-to-child transmission of fear and anxiety (also known as equifinality, Cicchetti & Rogosch, 1996). For example, parental reinforcement of child fear and avoidance can contribute to child fear and anxiety (Fisak & Grills-Tacquechel, 2007). Furthermore, one specific risk factor may lead to multiple outcomes (also known as multifinality, Cicchetti & Rogosch, 1996). Thus, an infant exposed to parental expressions toward novel stimuli may not necessarily acquire fear but have a different effect or it might not have any effect at all. Lastly, fears are most likely not a product of a single fear learning pathway, but a combination of multiple pathways (Muris & Field, 2011).

Previous reviews have concluded that modeling/vicarious learning is a significant contributing factor to child acquisition of fear and anxiety (Aktar & Bögels, 2017; Fisak & Grills-Taquelchel, 2007; Murray et al., 2009). However, these conclusions were based on narrative reviews; whereas, currently enough research has been done to carry out a meta-analytic review. Narrative reviews tend to lead to overly strong conclusions compared to systematic and meta-analytic reviews (Thomas-Odenthal et al., 2020). Importantly, conducting a meta-analysis allows us to quantify the size of the investigated effects. Previous reviews have also discussed the role of BI and parental anxiety (Fisak & Grills-Taquelchel, 2007; Aktar & Bögels, 2017), but their roles on infant fear and avoidance learning have not yet been systematically assessed. In the current meta-analysis, our first aim was to synthetize the evidence on the effect of infants’ modeling of parents’ anxiety on infants’ immediate fearful or avoidant reactions to novel stimuli in early life (between 6 and 30 months). Second, we aim to explore whether the effect of modeling is larger for temperamentally fearful infants (based on Susceptibility models, Belsky & Pluess, 2009; Ingram & Luxton, 2005; Nigg, 2006). Finally, we aim to explore if the effect of modeling on infants’ fear and avoidance is larger for infants with anxious parents (based on Murray et al., 2009). We expect that infants’ modeling of parents’ fearful expressions increase their fear and avoidance toward novel stimuli. Furthermore, we expect the effect of modeling to be stronger for behaviorally inhibited infants and infants of anxious parents.

## Methods

### Protocol and Registration

In this systematic review and meta-analysis, we followed PRISMA guidelines (Moher et al., 2009). Moreover, this meta-analysis was preregistered at OSF 10.17605/OSF.IO/XPRUS.

### Search Strategy

**Fig. 1:**
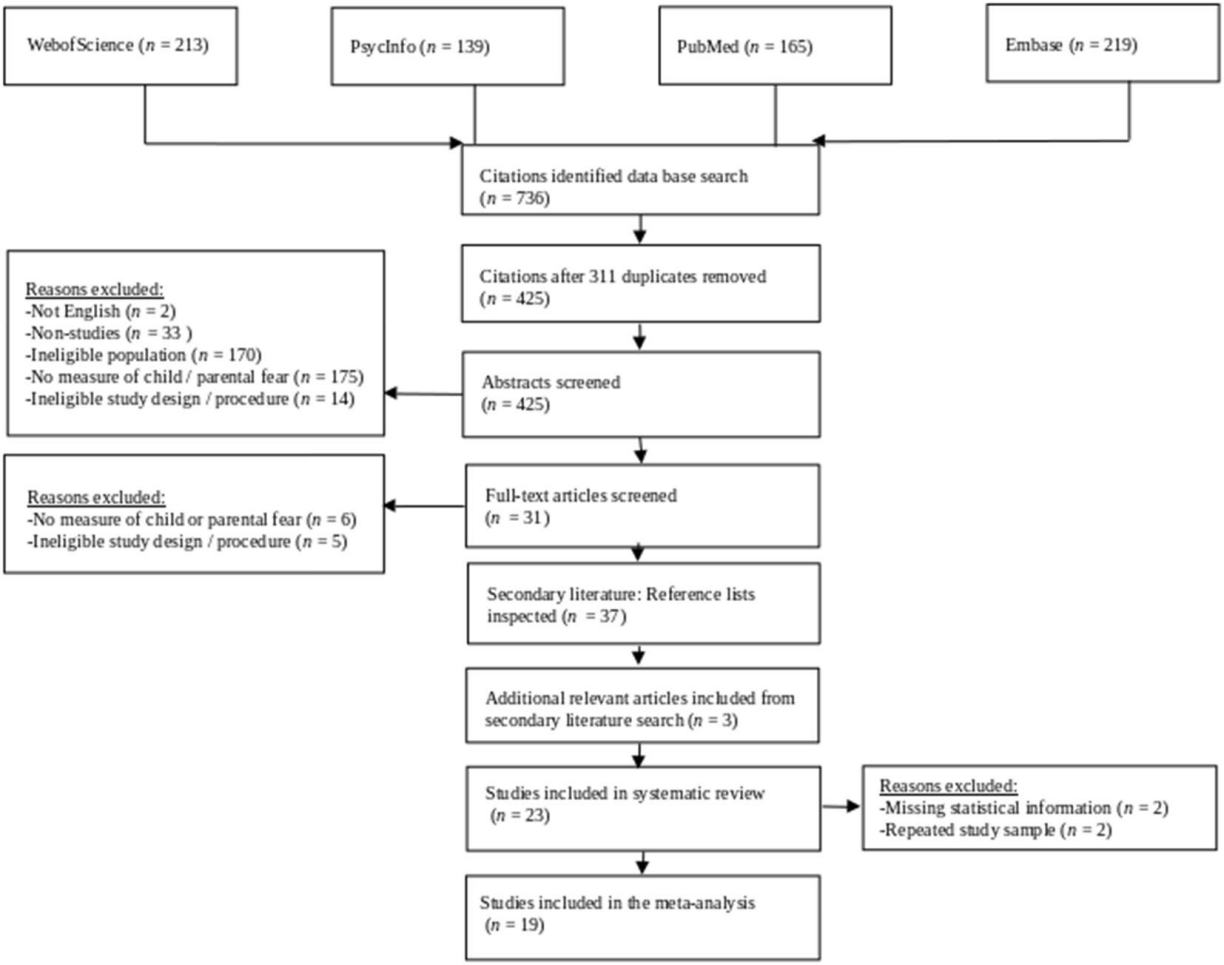
Flow Diagram

Web Of Science, Pubmed, Embase, and PsycINFO databases were searched to identify relevant articles. The search was performed on the 21st of November 2022. The final search term was ((postnat* OR neonat* OR newborn OR “new-born” OR infan* OR baby OR babies OR “month old” OR “month-old” OR toddler) AND (parent* OR mother* OR father* OR caregiver* OR guardian*) AND ((“social referencing” OR acquisition OR “nonverbal transmission” OR “non-verbal transmission” OR “vicarious learning” OR “observational learning”) AND (fear* OR avoid* OR anxi* OR threat*))). For a full overview of the development of the final search term used in this study, see the Full search term list in the Supplementary Material (A). All screening steps were conducted by two independent reviewers. The interrater agreement on the inclusion of studies during the abstract screening process was high, with Cohen’s kappa of 0.85. Inconsistencies between reviewers were discussed and resolved in coding meetings. The steps of the screening process are presented in Fig. 1.

### Inclusion Criteria

The systematic review and meta-analysis include published studies that assessed fearful or anxious expressions in human infants (aged up to 30 months) after direct exposure to parental fear or anxious expressions in a lab setting. The included studies tested how parents’ fear (in specific situations, toward an object, situation, or stranger) can shape their infants’ reaction to the same ambiguous situations, object, or stranger. Within the studies, it can also be named modeling/observational or vicarious learning (the study needs to include parental *non-verbal* expression of fear). The ambiguous stimuli, i.e., stranger, object, or situation, need to be novel. This means, for example, that the ambiguous object is an unfamiliar toy and that the infant has not played with or seen it previously. In addition, the fear/anxiety expression in front of the infant should be from the parent, not, for example, from an experimenter. The current meta-analysis included studies that assessed infant reactions with behavioral (i.e., crying), physiological (i.e., elevated heart rate), or cognitive (i.e., infant looks) measurements. We then categorized infant behavioral reactions into (1) infants’ affective response to stimuli (facial, bodily, or vocal expressions of fear, i.e., crying), as well as (2) avoidant response to stimuli (facial or bodily avoidance of stimulus, i.e., turning away from stimulus). Furthermore, the meta-analysis only included studies that assessed parental fearful or anxious expressions. Studies with an experimental design needed to include a target group, which was defined as infants who received fearful/anxious cues from the parent about a novel object, person, or situation. The control group needed to entail infants receiving neutral or positive cues given by the parent about a novel object, person, or situation. Studies have to be published in English. For the meta-analysis, the information provided in the results section of a given study should allow for the calculation of effect sizes for outcome measures.

### Data Extraction

The data that were extracted are demographic information (i.e., age of the participating parents and infants, gender, ethnicity, occupation/SES, and study location) and methodological characteristics (i.e., study design, number of outcome variables, measurement tools for predictor and outcome variables, and their validity). Furthermore, we extracted means, standard deviations, effect sizes, and corresponding 95% Confidence Intervals (CI) of the variables and associations of interest. Variables of interest are infant fearful or avoidant reactions, parent anxious/fearful expressions, parent psychopathology, infant temperament, and stimulus type (i.e., social versus non-social). All effect sizes were converted to Hedges’ *g*. In case multiple means and standard deviations were reported in a study, for instance, due to multiple outcome measures, we averaged the outcomes to yield a single study-wide effect size. In cases where insignificant findings were reported without providing further statistical information than the sample size and non-significance, we assumed a *p*-value of 0.5 (one-directional) to calculate the effect size, which results in an effect size of 0 with the accompanying variance (see Dusseldorp et al., 1999). This was done as excluding the insignificant finding from analyses would inflate the effect sizes. The effect sizes for the moderators were only investigated if a subset consisted of at least four studies (*k* ≥ 4) (Bakermans-Kranenburg et al., 2003).

### Statistical Analyses

Analyses were carried out using the metafor package in R. Statistical significance of the pooled SMD was assessed using a Z test at *p* < 0.05. We checked for heterogeneity using the *Q* test. A two-tailed *p* significance test was used with statistical significance with *p* < 0.05. To enable comparisons, calculated effect sizes were transformed into standardized scores. We corrected the effect sizes to a weighted effect size (corrected for unequal *n´*s) and checked for publication bias with funnel plots. In case of publication bias, a trim and fill method was applied. Furthermore, to detect outliers, we checked whether the standardized residuals were between 3.29 and − 3.29.

### Quality and Bias Assessment

The methodological quality of the retained articles was assessed using a checklist (presented in Table S1) based on the Cochrane Collaboration tool (ROB2) and adapted to our study design. Examples of these assessment criteria are the reliability of the outcome measures, as well as the transparency and selection of the reported results.

## Results

Our search term yielded overall 736 hits across Web of Science, PsycInfo, Pubmed, and Embase. After the removal of 311 duplicates, we ended up with 425 studies to screen. The screening process and reasons for exclusions at each stage are presented in the flow diagram (Fig. 1).

### Overview of Studies

The study characteristics of the studies included in the systematic review and meta-analysis are summarized in Table 1. The quality ratings ranged from 67 to 100%, with a mean percentage of 93% (for the quality rating per study see Table S1 in Supplementary Material B). Most studies randomized participants into the conditions and used reliable coding systems or measures. However, some studies did not adequately describe their hypotheses and/or reported more analyses than planned a priori.

**Table 1:** Overview of Studies included in Systematic Review

Study	General characteristics			Infant characteristics				Parent characteristics					
	Journal	Location	Design	*N *(*n* after exclusion)	% Girls	Range Mean age (months)	BI (%)	*N *(n after exclusion)	% Mothers	Mean age (years)	SES	Ethnicity % CAU	Anxiety (%)
Aktar et al. (2013)	Journal of Child Psychology and Psychiatry	Europe, The Netherlands	Correlational	122	54.92	12 (12.20)	Assessed with (LAB-TAB)	244 (242)	49.59	33.21	Moderate to High	93.03 (Parent)	31.15 (Current), 54.10 (Lifetime), Assessed with: ADIS
Aktar et al. (2014)	Journal of Child Psychology and Psychiatry	Europe, The Netherlands	Correlational	117 (subsample of Aktar et al., 2013)	54.7	30 (30)	Assessed with (LAB-TAB)	234 (232)	50.43	34	Moderate to High	92.31 (Parent)	55.56, Assessed with: ADIS
Aktar et al. (2018)	Journal of Clinical Child & Adolescent Psychology	Europe, The Netherlands	Correlational	122 (same sample as Aktar et al., 2013)	54.92	12 (12.20)	Assessed with (LAB-TAB)	244 (242)	49.59	33.21	Moderate to High	93.03 (Parent)	31.15 (Current), 54.10 (Lifetime), Assessed with: ADIS
Blackford and Walden (1998)	Infant Behavior & Development	North America, USA	Experimental	55 (42)	52.73	11–22	Assessed with IBQ and TBAQ	55 (42)	89	NA	Moderate	96 (Infant)	NA
Carpenter (2004)	Dissertation	North America, USA	Experimental	61 (58)	43.10	T1: 9 T2: 12 T3: 18	Assessed with IBQ	61 (58)	96.72	NA	Moderate to High	83.61(Infant)	NA
De Rosnay et al. (2006)	Behavior Research and Therapy	Europe, UK	Experimental	24 (24)	50	12–14 (12.8)	Assessed with IBQ	24 (24)	100	NA	NA	100 (Infant)	Low range for social anxiety
Dubi et al. (2008)	Journal of Abnormal Child Psychology	Oceania, Australia	Experimental	80 (71)	62	15–20 (17.39)	Assessed with Short Temperament Scale	80 (71)	100	NA	“well-educated”	94 (Infant)	Normal range, assessed with DASS
Gerull and Rapee (2002)	Behavior Research and Therapy	Oceania, Australia	Experimental	31 (30)	50	15–20 (17.16)	NA	31 (30)	100	NA	Moderate	87.5 (Infant)	NA
Goodman-Wilson (2012)	Dissertation	North America, USA	Experimental	94 (90)	47.87	12–13.5 (12.72)	Assessed with IBQ-R	94 (90)	100	NA	Moderate	71.28 (Parent), 63.83 (Infant)	Assessed with IAS and STICSA
Hirshberg & Svedja (1990)	Child Development	North America, USA	Experimental	74 (66)	50	12	NA	148 (132)	50	31	High	“Predominantly white” (Parents)	NA
Kim et al. (2010) (Study 1)	Infant Behavior and Development	North America, USA	Experimental	41(30)	60.98	17–25 (20.6)	NA	41(30)	90.24	NA	NA	92.68 (Infant)	NA
Klinnert (1984)	Infant Behavior and Development	North America, USA	Experimental	72 (11)	50	12—18, (15)	NA	72 (11)	100	NA	Moderate	NA	NA
Knieps et al. (1994)	American Journal of Mental Retardation	North America, USA	Experimental	22 (11, only subsample without Down Syndrome)	36.36	10–23 (16.2)	NA	22 (11)	90.91	NA	Moderate	90.91(Infant)	NA
Möller et al. (2014)	Developmental Science	Europe, The Netherlands	Correlational	81 (81)	50.62	10–15 (11.88)	Assessed with IBQ-R	81 (81)	49.38	35.62	High education level	NA	Assessed with SCARED-A, Mean: no anxiety
Mumme et al. (1996)	Child Development	North America, USA	Experimental	125 (90)	51.11	12–13 (12.43)	NA	125 (90)	100	NA	Moderate	81 (Infant)	NA
Murray et al. (2008)	Child Development	Europe, UK	Experimental	190 (156)	55.25	(T1 10.31, T2 14.04)	Assessed with behavioral coding of non-social tasks	190 (156)	100	30.80	Moderate to high	97.38 (Parent)	Assessed with SIAS, SPS, clinical structured interview for DSM IV, Social Phobia: 50.64%
Rosen et al. (1992)	Developmental Psychology	North America, USA	Experimental	39 (37)	48.65	11–12 (12)	NA	39 (37)	100	NA	Moderate	NA	NA
Sorce et al. (1985) (Study 1)	Developmental Psychology	North America, USA	Experimental	61 (36)	47.22	12	NA	61 (36)	100	NA	Moderate	NA	NA
Stenberg (2003)	Infant and Child Development	Sweden, Europe	Experimental	99 (96)	50	12.15	NA	99 (96)	100	31.9	High education level	100 (Infant)	NA
Walden and Ogan (1988)	Child Development	North America, USA	Experimental	40 (40)	45	6–22 (12.79)	NA	40 (40)	80	NA	Moderate	NA	NA
Walden and Baxter (1989)	Child Development	North America, USA	Experimental	48 (32 only age groups 6 to 23 months)	50	12.75	NA	48 (32 only parents of age groups 6 to 23 months)	85.42	NA	Moderate	NA	NA
Walden et al. (1991)	American Journal of Mental Retardation	North America, USA	Experimental	40 (20, only subsample without developmental delays)	45	6–27	NA	40 (20)	90	NA	Moderate	NA	NA
Zarbatany and Lamb (1985)	Infant Behavior and Development	North America, USA	Experimental	79 (18 in parent conditions)	Original sample: 48	Original sample 13.23–15.03 (14.13)	NA	18	100	NA	NA	NA	NA

For Study characteristics: Journal = name of journal in which the article was published; Location= study location. For Infant characteristics: *N* = number of infants in the sample; *BI* = behavioral inhibition/ temperament; *CAU* = Caucasian. For Parent characteristics: *N* = number of parents in the sample; *SES* = socio-economic status; *CAU* = Caucasian; *Anxiety* = percentage of parents who have anxiety based on diagnostic tools or questionnaires

### Systematic Review

The study and sample characteristics are presented in Table 1. The studies differed in (1) design, (2) moderators, (3) child fear index, (4) parental message type, and (5) stimulus type. Below we address each of these in detail.

First, concerning the design, from the 23 studies included in this systematic review four had a correlational design, whereas 19 had an experimental design. In the correlational designs, parental expressions of fear to novel stimuli were not manipulated/trained by the experimenter, but observed as it naturally unfolds during a social referencing paradigm with parents and their infants.

Second, of these studies, eight studies included a measure of parental anxiety symptoms or diagnosis. Four studies included clinical parent samples, consisting of 51% to 56% of parents with an anxiety disorder (Aktar et al., 2013, 2014, 2018; Murray et al., 2008), whereas four studies assessed anxiety (symptoms) in community samples of parents and reported no or low anxiety scores (De Rosnay et al., 2006; Dubi et al., 2008; Goodman-Wilson, 2012; Möller et al., 2014). Finally, ten studies of these 23 studies assessed infant temperament (Aktar et al., 2013, 2014, 2018; Blackford & Walden, 1998; Carpenter, 2004; De Rosnay et al., 2006; Dubi et al., 2008; Goodman-Wilson, 2012; Möller et al., 2014; Murray et al., 2008).

Third, there were also differences across studies, which child fear indices were assessed to test child acquisition of fear and avoidance (overview can be found in Table 2). Across all studies, infant fear was primarily assessed with a behavioral measure, specifically affective responses and avoidant responses toward the stimulus during the social referencing paradigm. In one study infant reactions were assessed with just a fear measure (i.e., facial, vocal, and verbal expressions of fear), in five studies only avoidance was assessed (i.e., latency touching and reaching for the toy), and in 17 studies both fear and avoidance were assessed. Looks to the caregiver were defined as an indicator of social referencing, rather than an index of infant fear. Nearly all studies reported mean interobserver reliability (ICC or Cohen’s kappa) for coding infant fear and avoidance (22 studies), which ranged from 0.56 to 1, and were all classified to be sufficient to very high interrater reliability.

**Table 2:** Overview of the reviewed studies’ approach to measuring parental non-verbal communication

Study	Type of parental message	Type of stimulus	Specifically	Assessment method	Specifically
Aktar et al. (2013)	Non-verbal and verbal	Social and non-social	Stranger and toy	Behavioral Measure: Fear and avoidance	Fear: Intensity and frequency of facial, bodily and vocal expressions of fear on a scale from 1 to 5 Avoidance: Infant attempt to gaze away, turn away or increase distance from or ignore stimuli on a scale from 1 to 5
Aktar et al. (2014)	Non-verbal and verbal	Social and non-social	Stranger and toy	Behavioral Measure: Fear and avoidance	Fear: Intensity and frequency of facial, bodily and vocal and verbal expressions of fear on a scale from 1 to 5 Avoidance: Infant attempt to gaze away, turn away hide from stimuli on a scale from 1 to 5
Aktar et al. (2018)	Non-verbal and verbal	Social and non-social	Stranger and toy	Behavioral Measure: Fear and avoidance	Fear: Intensity and frequency of facial, bodily and vocal expressions of fear on a scale from 1 to 5 Avoidance: Infant attempt to gaze away, turn away or increase distance from or ignore stimuli on a scale from 1 to 5
Blackford and Walden (1998)	Non-verbal and verbal	Non-social	Toy	Behavioral Measure: Fear and avoidance	Fear: Facial, vocal and verbal expressions of fear from 1 (very positive) to 5 (very negative) Avoidance: Approach and avoidance from stimulus 1 (approach) to 7 (avoidance)
Carpenter (2004)	Non-verbal and verbal	Social and non-social	Stranger and toy	Behavioral Measure: Fear and avoidance	Fear: Facial, bodily, and vocal expressions from 1 (extreme happiness) to 5 (extreme fear) Avoidance: Approach and avoidance from stimulus 1 (full approach) to 7 (full avoidance), Cognitive measure: looks to stimulus
De Rosnay et al. (2006)	Non-verbal and verbal	Social	Stranger	Behavioral Measure: Fear and avoidance	Fear: Facial, and bodily expressions of fear from 1 (absent) to 5 (very frequent) Avoidance: Attempts to increase distance from stranger by, i.e., gazing away, moving away, turning away from 1 (absent) to 5 (very frequent)
Dubi et al. (2008)	Non-verbal and verbal	Non-social	Toy	Behavioral Measure: Fear and avoidance	Fear: Emotional expression -2 (high negative affect) to 2 (high positive affect) Avoidance: Approach and avoidance from stimulus -2 avoidance (retreat to mother) to 2 approach (touching and exploring toy)
Gerull and Rapee (2002)	Non-verbal and verbal	Non-social	Toy	Behavioral Measure: Fear and avoidance	Fear: Emotional expression -2 (high negative affect) to 2 (high positive affect) Avoidance: Approach and avoidance from stimulus -2 avoidance (retreat to mother) to 2 approach (touching and exploring toy)
Goodman-Wilson (2012)	Non-verbal and verbal	Social and non-social	Stranger and toy	Behavioral Measure: Fear and avoidance	Fear: Facial and vocal expressions from 1 (very positive) to 5 (very negative) Avoidance: Approach and avoidance from stimulus, 1 (No avoidance) to 5 (strong avoidance)
Hirshberg & Svedja (1990)	Non-verbal and verbal	Non-social	Toy	Behavioral Measure: Fear and avoidance	Fear: Facial, bodily, and vocal expressions from 0 (no distress) to 2 (extreme distress) Avoidance: Number of approaches, time spent in approach, distance traveled in approach and latency to initiate approach in seconds
Kim et al. (2010)	Non-verbal and verbal	Non-social	Toy	Behavioral Measure: Avoidance (as inverse from Approach)	Avoidance: Proportion of time infant exhibited proximal toy-directed behavior
Klinnert (1984)	Non-verbal only	Non-social	Toy	Behavioral Measure: Avoidance	Avoidance: Farthest excusing toward toy, latency to approach toy
Knieps et al. (1994)	Non-verbal and verbal	Non-social	Toy	Behavioral Measure: Fear	Fear: Facial expression, verbalizations, and vocal tone from 1 (positive expression) to 5 (negative expression), 3 is neutral
Möller et al. (2014)	Non-verbal and verbal	Non-social	Visual cliff	Behavioral Measure: Fear and avoidance	Fear: Facial, vocal, and verbal expressions of fear on a 4-point scale from 0 to 3, indicating higher frequency Avoidance: Looking away, turning away, sitting still for a long time, on a 4-point scale from 0 to 3, indicating higher frequency,
Mumme et al. (1996)	Non-verbal only (also voice only but not used)	Non-social	Toy	Behavioral Measure: Fear and avoidance	Fear: Facial expression from 0 (neutral) to 2 (very negative) Avoidance: Turned away from toy, not approach toy at all (0) to approach/ interaction toy (3)
Murray et al. (2008)	Non-verbal and verbal	Social	Stranger	Behavioral Measure: Fear and avoidance	Fear: Intensity and frequency of facial, bodily, and verbal expressions of fear on a 5-point scale Avoidance: Infant attempt to gaze away, turn away or increase distance from stimuli on a 5-point scale
Rosen et al. (1992)	Non-verbal and verbal	Non-social	Toy	Behavioral Measure: Fear and avoidance	Fear: Assessed whether infant affective display was positive, negative, or unclear Avoidance: Distance from toy in meters
Sorce et al. (1985)	Non-verbal only	Non-social	Visual cliff	Behavioral Measure: Fear and avoidance	Fear: Intensity and frequency of facial, bodily, and verbal expressions of fear from 1 (smile) to 5 (overt distress) Avoidance (coping behavior): Presence or absence of crossing cuff, frequency of retreat back, moving to shallow side
Stenberg (2003)	Non-verbal and verbal	Non-social	Toy	Behavioral Measure: Fear and avoidance	Fear: Infant affect total number of 5-s intervals during which the infant showed positive, negative, and neural affect Avoidance: Amount of time averting gaze or turning away from toy, reaching for toy, time spent playing with toy
Walden and Ogan (1988)	Non-verbal and verbal	Non-social	Toy	Behavioral Measure: Avoidance	Fear: Frequency of crying Avoidance: Latency and frequency touching and reaching for toy
Walden and Baxter (1989)	Non-verbal and verbal	Non-social	Toy	Behavioral Measure: Avoidance	Avoidance: Latency touching and reaching for toy
Walden et al. (1991)	Non-verbal and verbal	Non-social	Toy	Behavioral Measure: Avoidance	Avoidance: Frequency, duration, and latency touching of toy
Zarbatany and Lamb (1985)	Non-verbal only	Non-social	Toy	Behavioral Measure: Avoidance	Avoidance: Latency to approach toy, distance moved toward toy

Fourth, parental expressions of fear toward novel stimuli can be categorized into (1) non-verbal messages only (such as fidgeting) and (2) non-verbal and verbal messages (such as “this is scary, right?”). Out of 23 studies, five studies fall in the first category, whereas 18 studies were in the second. Furthermore, in experimental designs, the threat condition was defined as fearful/anxious non-verbal messages (based on facial, bodily, or vocal expressions), whereas the control condition could either consist of parental neutral non-verbal expressions or positive non-verbal expressions (i.e., smiling). In 21 studies, the control condition in the social referencing paradigm consisted of positively valenced non-verbal parental messages and two studies included both a positive and neutral control condition (Klinnert, 1984; Mumme et al., 1996). Most studies reported mean interobserver reliability (ICC or Cohen’s kappa) for parent variables (17 studies), which ranged from 0.39 to 1, and all except for one (Zarbatany & Lamb, 1985) were classified as sufficient to very high.

Fifth, the stimuli that were paired with parental messages varied across studies and can be categorized into social and non-social stimuli. Social stimuli entailed exposure to a stranger, whereas non-social stimuli entailed animals, toys, and novel situations, such as a visual cliff. The majority of studies (*k* = 16) included non-social stimuli, whereas two studies used only social stimuli in their social referencing paradigms and five studies included both social and non-social stimuli.

### Meta-Analysis

For the meta-analysis, we only included studies that reported the statistical information that is necessary for the computing of effect sizes. We contacted authors for missing statistical information (such as missing sample sizes or standard deviations). We only received sufficient statistical information to analyze effect sizes from one study (Möller et al., 2014). We received three responses that the statistical information was not available (Blackford & Walden, 1998; Walden & Baxter, 1989; Zarbatany & Lamb, 1985) and one author did not respond (Klinnert, 1984). Furthermore, three studies that were included in the systematic review (Aktar et al., 2013, 2014, 2018) contained analyses of the same infants at different developmental stages. For the meta-analysis, we chose to include only data from the first study (Aktar et al., 2013), as it contained the largest sample size. Möller et al. (2014) reported their findings separately on a mother and father sample, which participated independently with different infants. Therefore, we added them as separate samples in our analyses.

Overall, of the 23 studies included in the systematic review, 19 studies entailing 20 samples were also included in the meta-analysis. Two studies had a correlational design (Aktar et al., 2013; Möller et al., 2014) and the remaining seventeen studies had an experimental design. Fourteen studies entailed non-social stimuli, two had only social stimuli, and three studies included both social and non-social stimuli. When a study measured infant acquired fear to both social and non-social stimuli, we combined the effect sizes (if relevant statistical information was available). Thirteen studies assessed infant fear/ anxiety with behavioral indices of fear and avoidance separately, three studies just assessed avoidance based on infant behavior, and one assessed only infant fear with a behavioral measure. Two studies assessed infant avoidance additionally with a cognitive measure. When we had multiple outcomes of fear or avoidance, we combined the effect sizes. If we could not combine indices, we chose the statistics in the following order: (1) behavioral measure of infant fear or avoidance and (2) cognitive measure of infant avoidance (such as frequency of looks). No study assessed physiological indices of fear.

Six studies that were included in the meta-analysis assessed parental anxiety, of which the sample of two studies consisted of 51% to 54% of parents with an anxiety disorder (Aktar et al., 2013; Murray et al., 2008). Four studies assessed the absence of an anxiety disorder/symptoms (De Rosnay et al., 2006; Dubi et al., 2008; Goodman-Wilson, 2012; Möller et al., 2014). However, only three studies that were included in the meta-analysis of main effects reported findings on parental anxiety as a moderator, and therefore we could not perform analyses on its effect size. Eight studies that were included in the meta-analysis assessed infant temperament and reported relevant statistical information (Aktar et al., 2013; Carpenter, 2004; De Rosnay et al., 2006; Dubi et al., 2008; Goodman-Wilson, 2012; Möller et al., 2014; Murray et al., 2008).

### Main Results

#### Meta-Analysis

The effect of parental threat expression on infant *fear* was Hedges’ *g* = . 39, *SE* = 0.13, *CI* [0.14, 0.64], *k* = 17, *p* < 0.01), indicating that infants displayed more fear toward the novel stimulus after being exposed to parental threat expressions. There was an indication of heterogeneity (*Q* = 76.50, *p* < 0.0001). Egger’s test did not indicate asymmetry in the funnel plot (*b* = − 0.03, *p* = 0.34), and the trim-fill method did not indicate missing studies on the left side of the funnel. In a sensitivity analysis, we repeated the same analysis with only experimental studies. In experimental studies, the effect size of parental threat expression on infant *fear* was Hedges’ *g* = . 44, *SE* = 0.15, *CI* [0.14, 0.73], *k* = 14, *p* < 0.01), with no indication of funnel plot asymmetry (*b* = -0.11, *p* = 0.32) or missing studies on the left side of the funnel.

**Fig. 2:**
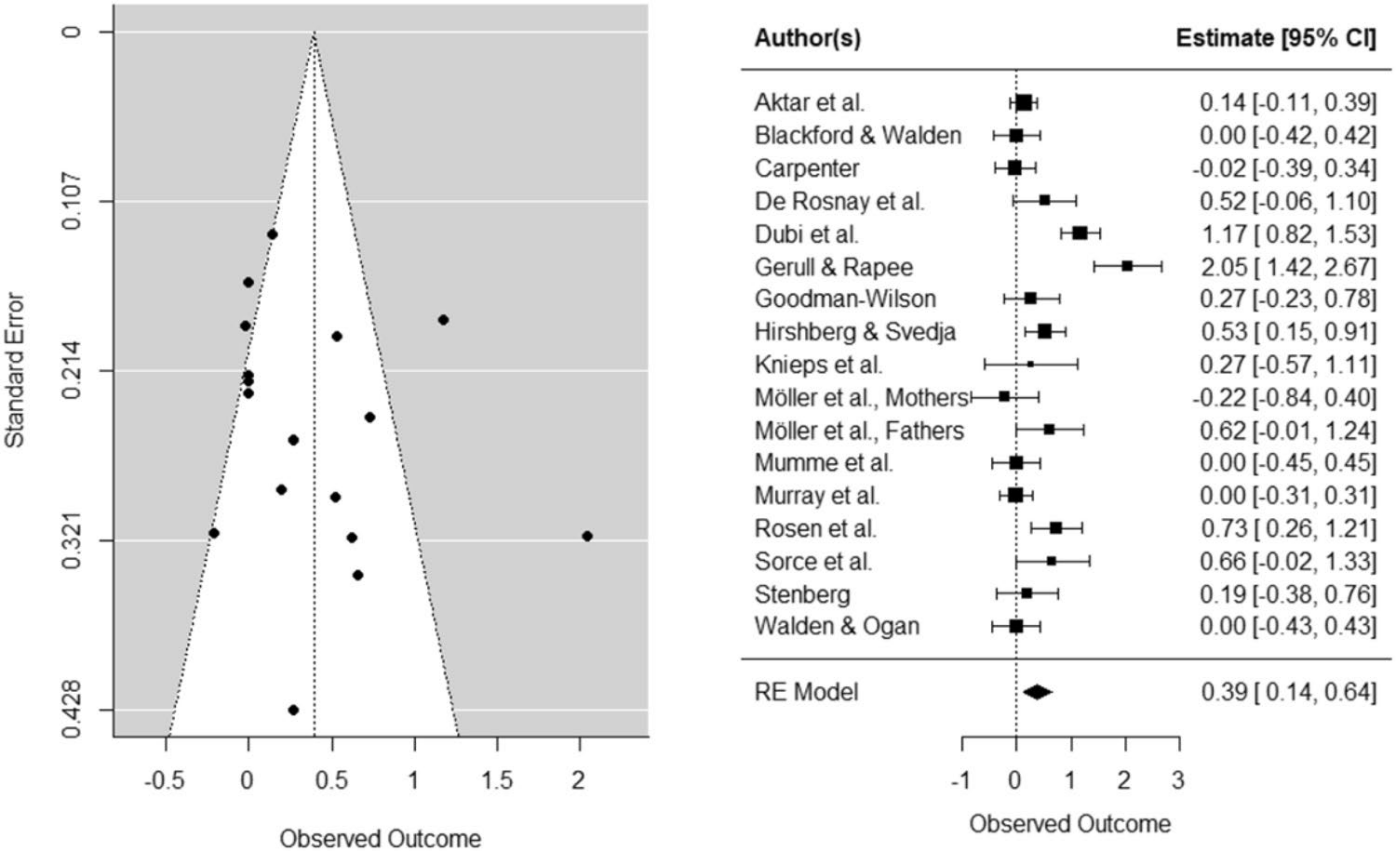
Funnel and forest plots of main effects on child fear

**Fig. 3:**
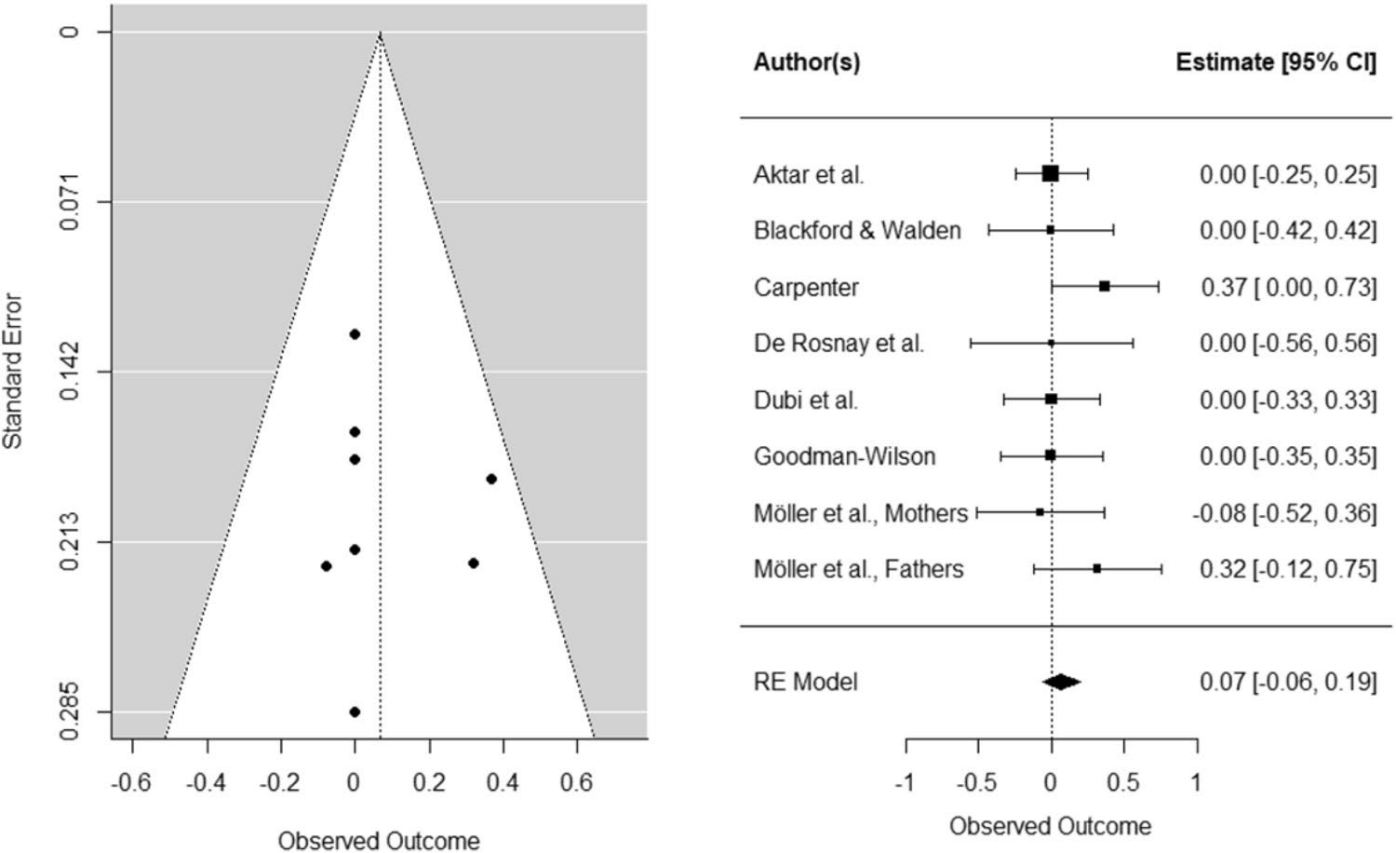
Funnel and forest plots of moderating BI effect on child fear

The effect with infant *avoidance* as an outcome measure was Hedges’ *g* = 0.46, *SE* = 0.10, *CI* [0.26, 0.65], *k* = 19, *p* < 0.0001), indicating that infants were more avoidant of the novel stimulus after being exposed to parental threat expressions. There was an indication of heterogeneity (*Q* = 52.49, *p* < 0.0001). Egger’s test did not indicate asymmetry in the funnel plot (*b* = 0.04, *p* = 0.18), and the trim-fill method did not indicate missing studies on the left side of the funnel. In experimental studies, the effect size of parental threat expression on infant *avoidance* was Hedges’ *g* = . 44, *SE* = 0.12, *CI* [0.21, 0.68], *k* = 16, *p* < 0.01), with no indication of funnel plot asymmetry (*b* = 0.03, *p* = 0.30) or missing studies on the left side of the funnel. For both fear and avoidance outcomes, funnel and forest plots can be found in Figs. 2 and 3 (for plots of studies with only experimental design see Supplementary Material C). Inspection of the standardized residuals revealed no outlier (all standardized residuals between 3.29 and − 3.29). Lastly, we checked whether study effect sizes for infant fear or avoidance were related to the study quality ratings, which was not the case (both *p’*s > 0.67).

### Systematic Review

A summary of the main findings can be found in Table 3. Based on social fear learning theories (Olsson et al., 2007; Rachman, 1977), we expected that infants express more fear and anxiety toward novel stimuli when these stimuli are paired with parents’ fear/anxiety expressions than non-anxious parental expressions. Of the 23 studies reviewed, infant fear was assessed in 17 studies, infant avoidance was assessed in 21 studies, and a combined fear and avoidance measure was utilized in two studies. We found that six out of 17 (44%) studies found an effect of parental expressions of fear/anxiety on infant fear (measured as infant fearful/negative affect/distress) and the other eleven did not. Furthermore, 11 out of 21 (52%) studies found an effect on infant avoidance (Aktar et al., 2013; De Rosnay et al., 2006; Dubi et al., 2008; Gerull & Rapee, 2002; Hirshberg & Svedja, 1990; Kim et al., 2010; Mumme et al., 1996; Sorce et al., 1985, Walden & Ogan, 1988; Walden & Baxter, 1989; Walden et al., 1991), one study found an effect on avoidance of toys but not of the stranger (Goodman-Wilson, 2012), and another only on part of the stranger task (pick up but not approach phase) (Murray et al., 2008). Lastly, one of the two studies that included a combined fear and avoidance measure found no relationship between parental non-verbal signals of threat and infant reaction to novel stimuli (Aktar et al., 2014), whereas the other one did (Rosen et al., 1992).

**Table 3:** Main outcomes and results of moderators on the association between parental non-verbal communication and infant fear/avoidance outcomes

Study	Main Outcomes Moderator Outcomes	
		Behavioral Inhibition
Aktar et al. (2013)	Fear: — Avoidance:↑	Fear: — Avoidance: ↑
Aktar et al. (2014)	Fear/Avoidance: —	Fear/Avoidance: —
Aktar et al. (2018)	Fear: — Avoidance: —	NA
Blackford and Walden (1998)	Fear: — Avoidance: —	Fear: — Avoidance: —
Carpenter (2004)	Fear: — Avoidance: —	Fear: ↑ Avoidance: —
De Rosnay et al. (2006)	Fear: ↑ Avoidance: ↑	Fear: — Avoidance: ↑
Dubi et al. (2008)	Fear: ↑ Avoidance: ↑	Fear: — Avoidance: —
Gerull and Rapee (2002)	Fear: ↑ Avoidance: ↑	NA
Goodman-Wilson (2012)	Fear (Stranger and Toy): — Avoidance (Stranger and Toy): —	Fear (Stranger and Toy): — Avoidance: Stranger ↓Toy —
Hirshberg & Svedja (1990)	Fear: ↑ Avoidance: ↑	NA
Kim et al. (2010)	Avoidance: ↑	NA
Klinnert (1984)	Avoidance: —	NA
Knieps et al. (1994)	Fear: —	NA
Möller et al. (2014) Mother Sample	Fear: — Avoidance: —	Fear: — Avoidance: —
Möller et al. (2014) Father Sample	Fear: ↑ Avoidance: ↑	Fear: — Avoidance: ↑
Mumme et al. (1996)	Fear: — Avoidance: —	NA
Murray et al. (2008)	Fear: — Avoidance: —	NA
Rosen et al. (1992)	Fear/Avoidance: ↑	NA
Sorce et al. (1985)	Fear: ↑ , Avoidance: ↑	NA
Stenberg (2003)	Fear: — Avoidance: —,	NA
Walden and Ogan (1988)	Fear: — Avoidance: ↑	NA
Walden and Baxter (1989)	Avoidance: ↑	NA
Walden et al. (1991)	Avoidance: ↑	NA
Zarbatany and Lamb (1985)	Avoidance: —	NA

↑ = increase in (or presence of) non-verbal communication significantly associated with increase in or higher fear/anxiety *p* < .05;

— = non-verbal communication not significantly associated with fear/anxiety *p* > .05, if main effect insignificant but 3 -or 4-way interaction significant it is labeled as insignificant;

NA = interaction not assessed (i.e., only main effect and not interaction with parental fear expression or 3-way interactions with another variable) or not assessed at relevant time point/age range

Multiple studies investigated additional moderating effects of for example parental gender, infant gender, and/or age in the link between parental-expressed fear and infant fear/avoidance to novel stimuli. In one study, authors found an effect of parental threat on infant fear and avoidance, but only when the father conveyed the fearful signals and not the mother (Möller et al., 2014), whereas another study found only maternal, but not paternal expression being related to subsequent infant fear when the infant was one year old (Aktar et al., 2018). Carpenter (2004) found an effect of parental threat on infant fear and avoidance only in specific age ranges. Nine-month old’s looked less to stimuli in fear vs happy condition and 18-month old’s showed overall less approach in fear versus happy condition, whereas Walden and Baxter (1989) found an effect in the 13- to 23-month-olds but not in 6–12- or 24–40-year old’s. Another study found mothers’ messages to only affect female infants, who stayed less close to the toy in the fearful versus happy condition (Rosen et al., 1992).

### Parental Anxiety and Child BI

#### Meta-Analysis

BI was not a significant moderator of infant *fear*. The effect of parent responses on infant fear did not change as a function of BI (Hedges’ *g* = 0.07, *SE* = 0.07, *CI [*− 0.06, 0.19], *k* = 8, *p* = 0.31. In a sensitivity analysis, we repeated the same analysis with only experimental studies. Again, the effect size of parent responses on infant fear did not change as a function of BI, Hedges’ *g* = . 08, *SE* = 0.09, *CI* [− 0.09, 0.25], *k* = 5, *p* = 0.36).

BI was a significant moderator on infant *avoidance*: the effect of parent responses was stronger for infants higher in BI (Hedges’ *g* = 0.25, *SE* = 0.11, *CI* [0.04, 0.46], *k* = 8, *p* < 0.05). In a sensitivity analysis, we repeated the same analysis with only experimental studies. In contrast to findings including both correlational and experimental studies, the effect size of parent responses on infant fear did not change as a function of BI when solely including experimental studies (Hedges’ *g* = 0.18, *SE* = 0.13, *CI* [− 0.07, 0.32], *k* = 5, *p* = 0.17).

**Fig. 4:**
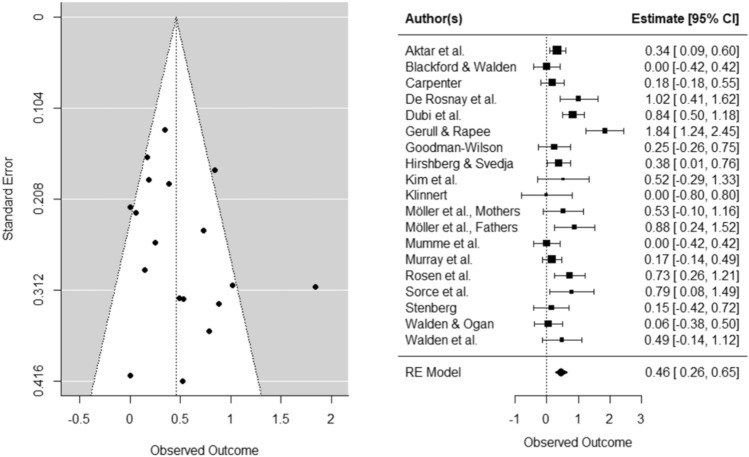
Funnel and forest plots of main effects on child avoidance

**Fig. 5:**
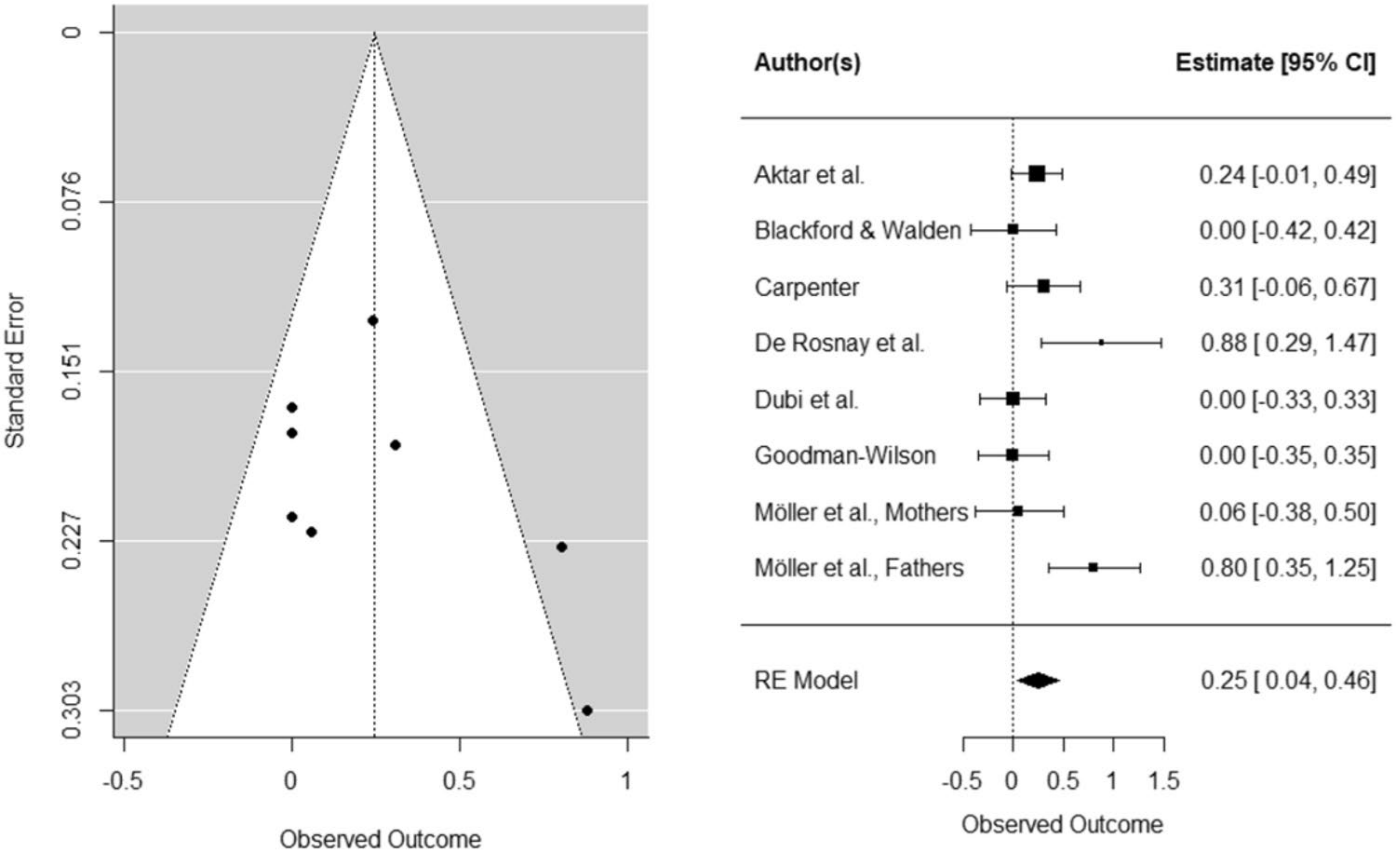
Funnel and forest plots of moderating BI effect on child avoidance

Funnel and forest plots can be found in Figs. 4 and 5 (for plots of studies with only experimental design see Supplementary Material C). Inspection of the standardized residuals revealed no outliers. We could not assess whether parental anxiety moderates the effect of parental responses on infant fear and avoidance because we did not have enough studies for the analysis (*k* < 4).

#### Systematic Review

A summary of the moderator effects can be found in Table 3. Of the 23 studies reviewed, only three assessed the moderating role of parental anxiety (Aktar et al., 2014; Goodman-Wilson, 2012; Murray et al., 2008). The study by Aktar et al. (2014) found that the link between parental expressions of threat at 12 months with infant fear/avoidance at 30 months was stronger for infants of parents with lifetime comorbid social and other anxiety diagnoses. However, the study did not assess the moderating role of parental anxiety in the link between parental expressions of threat at 12 months with infant fear/avoidance on the same day. Goodman-Wilson (2012) did not find a significant moderating effect on infant fear or avoidance. Murray et al. (2008) found that infants of mothers with social phobia at 10 months were not more avoidant toward or fearful of strangers in the same social referencing paradigm. However, they did become more avoidant of strangers picking them up (but not approaching them) between 10 and 14 months. Thus, no study found that the effect of parental anxious expression on infant fear or avoidance was stronger in infants of anxious parents, when assessing the outcome in the same social referencing paradigm.

We expected that infants with a more fearful temperament express more fear and avoidance of novel stimuli than less temperamentally fearful infants when they are exposed to parental-expressed fear. Based on the 23 studies reviewed, we found some support for the hypothesis. Two out of eight studies (Aktar et al., 2013; De Rosnay et al., 2006) found a moderating effect of BI on infant *avoidance*, while five did not find such an effect (Blackford & Walden, 1998; Carpenter, 2004; 1998; Dubi et al., 2008; Möller et al., 2014) and one study (Goodman-Wilson, 2012) found an effect when the stimulus was a social but not non-social task (but in the opposite direction): Infants who were *low* in BI showed increased avoidance after parents expressed fear toward the stranger. However, Möller et al. (2014) did find a moderating role of infant BI on the impact of parental fear signals on infant avoidance of novel stimuli when fathers were conveying the fearful message and not mothers. The link between *paternal* fearful expressions and infant avoidance of the novel stimulus was stronger for infants with more fearful temperaments. In addition, while 1 study did find infant BI to impact the effect of parental fear signals on infant *fear* toward novel stimuli (Carpenter, 2004), the majority of studies (7 out of 8) assessing the moderating role on infant *fear* did not find an effect (Aktar et al., 2013; Blackford & Walden, 1998; De Rosnay et al., 2006; Dubi et al., 2008; Goodman-Wilson, 2012; Möller et al., 2014; Murray et al., 2008.)

## Discussion

This systematic review and meta-analysis aimed to shed light on the role of modeling in the transmission of fear from parents to infants. The meta-analytic evidence reveals that parental fear expressions to novel stimuli increase infant fear and avoidance—after a single exposure to these stimuli (Hedges’* g* = 0.44 and 0.44, respectively). We did find that behaviorally inhibited infants had stronger avoidance (not fear) reactions toward novel stimuli after exposure to parental fear expressions when including experimental and correlational studies, but the effect did not hold in experimental studies only. Below, we address each of these findings in turn.

### Infant Fear and Avoidance

In line with social fear learning models (Olsson et al., 2007; Rachman, 1977), there was a significant effect of parental fear expressions on infant fear and avoidance, supporting the idea of parental modeling as a social fear learning pathway with small to medium effect sizes (Hedges’* g* = 0.39 and 0.46, respectively). When investigating the causal effect of parental modeling on infant fear and avoidance using exclusively experimental studies, the effect sizes were also small to medium (Hedges’* g* = 0.44 and 0.44, respectively).

While experimental designs allow us to make stronger inferences on causality, their findings might be less generalizable to real-life interactions/daily life (Kazdin, 2021). A previous review that summarized findings on child fear acquisition via verbal threat information has argued for assessing child fear acquisition with this social fear learning paradigm in more ecologically valid contexts (Muris & Field, 2010). This reasoning also applies to modeling pathways in early life, as both experimental as well as prospective, more naturalistic designs are necessary to gain further insight on the impact of parental fear/anxiety expressions on infant fear and avoidance acquisition toward novel stimuli.

Furthermore, there seem to be inconsistent findings regarding infant fear and avoidance. Some studies that assessed both fear and avoidance toward novel stimuli as separate constructs, found different results for fear and avoidance outcome measures in the same children (i.e., support for an effect of condition on avoidance but not infant affect) (for example, Walden & Ogan, 1988). The mixed findings suggest that infant affect and avoidance do not have to co-occur, and different behavioral indices of fear might become relevant at different developmental stages. Infants’ ability to understand and judge how threatening a novel stimulus is, as well as to what extent they can regulate emotions accordingly can influence how infants express fear (LoBue & Adolph, 2019). During childhood, over time the inclination to avoid novel stimuli increases more strongly than to show distress (Rapee & Spence, 2004; Sumter, et al., 2009). As infants get older, infants may also show fewer fearful and more avoidant strategies in response to parental expression of fear (Aktar et al., 2018). Longitudinal studies covering the periods including and extending from the toddlerhood years are needed to shed light on these developmental differences in fearful and avoidant responses.

### Parental and Child Anxiety Dispositions

Susceptibility models suggest that infants with fear-sensitive temperamental dispositions are more susceptible to exposure to environmental stressors, including parental anxiety signals (Belsky & Pluess, 2009; Ingram & Luxton, 2005; Nigg, 2006). In line with this model, our meta-analysis suggests that there is a small moderating effect of BI on infant *avoidance* (Hedges’* g* = 0.25). Also the systematic review lends some support to the idea that behaviorally inhibited infants display an increased avoidant response after exposure to parental fear expressions, but only in three out of eight studies. However, based on the experimental studies we did not find a moderation by temperament in the causal link between parental fearful expressions to novel stimuli and child avoidance (Hedges’* g* = 0.18). Possibly, infants are more likely to display their usual/learned responses in response to more naturalistic parental expressions of fear (in correlational studies), than manipulated fear expressions (in experimental studies). Given that temperamentally fearful infants have the trait tendency to withdraw and avoid novel stimuli (Stifter & Augustine, 2019), this effect might be more visible in studies with correlational designs. Possibly, a third variable that is related to both parental fear expressions and infant avoidance may have inflated the effect size of this link. The reported correlations could, for example, be influenced by genetic similarity or the learning history of parental behaviors. Hence, we do not know to what extent the link between parental fear expressions to novel stimuli and child fear or avoidance is due to genes or to what extent it represents the habitual reaction of infants that has been reinforced over time. Specifically, parents may have supported children’s avoidant behaviors in anxiety-inducing or novel situations or removed the child from these situations (Reinforcement pathway described in Fisak & Grills-Tacquechel, 2007). In experimental studies, these confounds are controlled for by manipulating parental expressions/reaction. However, in experimental studies, the findings might also not be representative of the effect that parental fear expressions have on child fear and avoidance in real life, because manipulated parental expressions/reaction to novel stimuli might be different to infants’ previous experiences and expectations. A previous study suggests that expectancy violations in infants might influence social learning processes (Colomer & Woodward, 2023). It is up to future research to elucidate the role of BI in the parent-to-infant transmission of fear to novel stimuli by assessing the additional effects of genetic traits or assessing the influence of BI on the repeated exposure to parental fearful expressions.

Moreover, our meta-analysis did not find a moderating effect of BI on infant *fear* (Hedges’ *g* = 0.07). This aligns with findings from our systematic review where no such effect on infant fear was observed for the majority of the studies (7 out of 8 found no significant effect). Although this finding is not in line with the susceptibility theory (Belsky & Pluess, 2009), it might be explained by the fact that BI is most relevant in fear acquisition regarding social stimuli. BI is a more prominent risk factor for social anxiety (Clauss & Blackford, 2012) than for specific phobias (Pérez-Edgar & Fox, 2018), and in our systematic review most studies finding an effect included social stimuli in their social referencing design. Given that the majority of the included studies assessed fear toward non-social stimuli, no firm conclusions can be drawn on the moderating role of BI in the context of fear acquisition regarding social stimuli. Future experimental studies that incorporate social stimuli in their design will help to clarify this.

Next, we investigated the moderating role of parental anxiety. In the absence of a sufficient amount of studies with statistical information regarding parental anxiety, we could only assess its influence on infant fear and avoidance of novel stimuli by means of a systematic review. While parental anxiety is one of the biggest risk factors for child anxiety, we did not find support of infants of anxious parents showing stronger fear acquisition via modeling parents’ anxious expressions. This suggests that infants of anxious parents might not be more sensitive toward novel stimuli in the context of a single exposure to parental fear expressions. However, two studies, which did not find stronger fear or avoidance in the infants of anxious parents immediately after being exposed to parent’s expression of fear, found these expressions to be predictive of later avoidance toward that stimulus (Aktar et al., 2014; Murray et al., 2008). Specifically, Murray et al. (2008) found that infants of mothers with social phobia did become more avoidant of strangers picking them up between 10 and 14 months. The study by Aktar et al. (2014) found that the link between parental expressions of threat at 12 months with infant fear/avoidance at 30 months was stronger for infants of parents with lifetime comorbid social and other anxiety diagnoses. Therefore, it could be that over time, the repetitive nature of infant’s modeling of parental expressions of fear in families with anxious parents, entailing a higher frequency of parental anxious expressions to novel stimuli, could explain the familial aggregation of anxiety. Furthermore, anxious parents may be more likely to support infants’ avoidant behaviors in anxiety-inducing or novel situations or remove the infant from these situations/stimuli (Fisak & Grills-Tacquechel, 2007). Anxious parents might also provide adaptive emotion regulation strategies less frequently, such as offering a security object, or displaying alternative problem-solving behaviors (Stifter & Augustine, 2019). Moreover, they might also react unsupportive, for example, dismissing or ignoring infants’ emotional reactions. This in turn could decrease infants’ feelings of self-efficacy for self-regulation and increase the distress or fear response (Stifter & Augustine, 2019). Given the limited number of studies investigating the moderating role of parental anxiety in parent–infant fear transmission, we need more studies investigating its role.

### Clinical Implications

Heightened offspring fear or fear learning in response to (potentially) threatening stimuli represents an evolutionary-adaptive and normative process (Kiel & Kalomiris, 2019). Nevertheless, by understanding how social fear learning processes unfold and differ between healthy and at-risk families, we might eventually shed more light on the specific processes and factors to target in prevention and treatment efforts. In our study, we found a small to medium effect of parents displaying fearful reactions to novel stimuli on infant fear and avoidance toward these stimuli, independent of parental anxiety levels. While this fear acquisition pathway in itself can be seen as an adaptive response to threatening/novel situation, it does not exclude the possibility that in at-risk families, where exposure to parental anxious expressions in daily life can be more frequent or intense, the impact of this fear acquisition pathway can be amplified. Incorporating psychoeducation targeting the potential pathways of social fear transmission in parents and children might be helpful in the prevention of anxiety risk in the offspring. Given that the effect of parental fearful reactions to novel stimuli on infant avoidance was stronger for children with more fearful temperaments—psychoeducation might be valuable for parents with children who are behaviorally inhibited.

In real life, infants might not only get exposed to a fearful reaction of one (anxious) parent in isolation, but another parent or significant other may display the same or conflicting emotional responses. As fear modeling seems to lead to an infant’s fear acquisition toward novel stimuli, modeling of parents’ positive emotions or confident reactions may reduce or prevent fear acquisition, even when one parent displays anxious responses. This was recently summarized in a systematic review investigating whether infants and children’s positive modeling (of parents, strangers, and peers) in experimental, non-clinical contexts can reduce/prevent acquired fears (Krause & Askew, 2022). Although their conclusions rely on a limited amount of studies, positive modeling seems to be a promising technique to prevent fear acquisition and reduce fear responses in infants and children. Understanding how fears are acquired in developmentally sensitive designs can inform us of potential strategies to reduce or prevent parent-to-child fear transmission in at-risk families.

### Limitations and Future Directions

This is the first systematic review and meta-analysis on the effect of parental fear expressions on infant fear and avoidance of novel stimuli. Although this work provides a relatively less biased synthesis of available evidence on parent–infant fear transmission via modeling, this study is not without shortcomings and echoes the limitations of the singular empirical work. First, in our systematic review and meta-analysis we heavily relied on studies with WEIRD (Western, educated, industrialized, rich, and democratic) samples. It is important to acknowledge the role of cultural differences in the emotional development of infants since parents’ emotional expressions during daily interactions are part of commonly shared socialization practices (Halberstadt & Lozada, 2011). For example, infant’s attention to (parental) emotional expressions in daily life can vary depending on their socio-economic status (SES) (Clearfield & Jedd, 2013). Regarding our meta-analysis, this means we cannot generalize our findings to non-WEIRD samples. Future research that replicates previous studies in new or more heterogeneous samples, or compare the fear acquisition pathway across different cultural environments, can give us more insight on the generalizability of our findings (Nielsen et al., 2017).

Second, studying fear modeling in strict experimental lab designs allows stronger conclusions, but it restricts the ecological validity of the findings. In daily life, infants are usually exposed to ambiguous stimuli, such as novel toys in their own home or daycare, surrounded by familiar people, instead of in a new and ambiguous place with strangers (i.e., a lab, which does not characterize their common experience). Also training the parents to show specific emotional expressions in lab settings may not capture the intensity that the parent in real-life displays to novel stimuli. Furthermore, infants might not only get exposed to a fearful reaction of one parent in isolation, but often the two parents or significant others display similar or conflicting emotional responses, either simultaneously or successively. More research is needed to investigate fear modeling in multiple contexts, as well as naturalistic observations in clinical samples. Future studies might also investigate repeated exposure to parental fearful expressions (either via experimental manipulation or by inclusion of anxious parents) to examine whether repeated exposure predicts fear or avoidance to novelty over time and whether the relationship becomes stronger. This might also represent real life more accurately, as infants most likely will not only get exposed to parental expression to a novel stimulus just once.

Another limitation concerns the fact that multiple studies measured fear as a behavioral response to novel stimuli by exclusively focusing on either fear *or* avoidance. Studies focusing on singular indices of fear may not be sufficient to capture the entirety of infant fear reactions and do not allow us to investigate relationships between different fear indices. Measuring fear with singular indices can increase the likelihood of falsely identifying an infant’s reaction as fear (LoBue & Adolph, 2019). Therefore, to decrease the chances of misattribution, measuring fear in infants should contain multiple complementary methods, such as multiple behavioral (infant distress and avoidance) and physiological indices of fear (LoBue & Adolph, 2019). This would also allow us to investigate whether parental fear expressions influence various behavioral but also physiological reactions in infants. Moreover, in a longitudinal design, one could also assess which indices of infant fear can predict later development of fear or anxiety to novel stimuli.

## Conclusion

To conclude, we found a small to medium effect of parental fear signals toward novel stimuli on infant fear and avoidance of the stimuli—after a single exposure to that stimulus. Parents’ non-verbal reactions to novel stimuli matter and contribute to infant fear and avoidance learning. The infants’ levels of behavioral inhibition might increase avoidance to novel stimuli after exposure to parental expressions of fear, but more research is needed to conclude whether infant behavioral inhibition strengthens early environmental acquisition of fears and avoidance via parental modeling.

## Supplementary Information

Below is the link to the electronic supplementary material.

Supplementary file1 (DOCX 672 KB)
