# Supplementary material for: Parent to Offspring Fear Transmission via Modeling in Early Life: A Systematic Review and Meta-Analysis
Source: Clin Child Fam Psychol Rev. 2023 Jul 27;26(3):751–72. doi: 10.1007/s10567-023-00448-1 (PMC10465674; doi:10.1007/s10567-023-00448-1)

**Supplementary Material**

**A**

*Search strategy*

To identify relevant articles, WebOfScience, PUBMED, Embase, and PsycINFO databases were searched. The search will include studies up to 21-11-2022 in English. The search term was created after searching for synonyms in dictionaries and in previous literature for the key components: “infant”, “parent”, “fear” and “vicarious learning”. The resulting terms were combined, and the search term was adjusted. The initial proposed search term was: (("postnat*" OR "neonat*" OR "newborn" OR "new-born" OR "infan*" OR "baby" OR "babies" OR "month* old" OR toddler*) AND (parent* OR mother* OR father* OR caregiver* OR guardian*) AND ("social referenc*" OR acquisit* OR learn*) AND (fear* OR avoid* OR anxi* OR threat*)). We decided to shorten this for the following reasons: 1) we wanted to combine the vicarious learning and fear terms in order to find results more specific and relevant to our field of study, and exclude medical studies about viral/ bacterial transmission, and 2) this search term provided a large number of results, many of which were medical or animal studies, which are not relevant to our study. The final search term was: ((postnat* OR neonat* OR newborn OR "new-born" OR infan* OR baby OR babies OR "month old" OR "month-old" OR toddler) AND (parent* OR mother* OR father* OR caregiver* OR guardian*) AND (("social referencing" OR acquisition OR "nonverbal transmission" OR "non-verbal transmission" OR "vicarious learning" OR "observational learning") AND (fear* OR avoid* OR anxi* OR threat*))).

**B**

*Quality assessment*

Domain 1 - *Randomization process*

*In case of one condition:* Were all subjects selected or recruited from the same or similar population? (e.g. same age range and healthy sample)

*In case of control vs. experimental condition:* Were participants randomized into conditions/ or were groups matched based on age and sex? (if not randomized).

No (0): Not a similar population or not matched or unclear

Yes (1): Participants were randomized into conditions, they matched the groups before the group allocation *or* they checked if groups did not differ and they were similar *or* they checked if groups did not differ and they controlled for the variables that were not similar

Domain 2 - *due to deviations from intended interventions*

Did the stimuli remain novel for the participants?

No (0): if participants already had some interaction with the stimulus beforehand

Yes (1): if stimulus remained novel (e.g.: making sure stranger is not seen before experiment)

1. For social stimuli: Did the experimenter(s) make sure that stranger(s) were not aware of conditions?

No (0): the randomized allocation was not concealed; thus it is likely that strangers (who deliver the intervention) were aware of participants' assigned intervention during the trial (e.g. if stranger sees babies allocation into experimental and control group beforehand)

Yes (1): the experimenters made sure that strangers were not able to see any part of the randomized allocation of the babies (= they remained blind)

Domain 3 -*Missing Data*

Was there less than 20% of dropout? (or if there is none mentioned but no way of checking it - to answer this question check if they mention the sample size in the descriptives and check if this number of participants is the same in the analyses and check if they have a paragraph about missing data and check if there was less attrition than 20%)

No (0): there was more than 20% attrition, *or* they have not mentioned how much attrition they specifically had

Yes (1): there was less than 20% of attrition

Domain 4 – *Measurement of outcome*

1. For observed infant anxiety/fear/avoidance: was the measurement of this outcome variable reliable?

- No (0): the ICC (inter-coder reliability) was <.60 *or* in the text there was something like ‘low reliability’ *or* they do not mention something about reliability at all
- Yes (1) = the reliability for the outcome measure is higher than >.60
- States that for behavioral response was excellent, however for emotional response moderate – count as yes (1)

1. For child temperament (e.g. behavioral inhibition, general fearfulness, fearful temperament)/ parental anxiety only: In case there was behavioral inhibition or parental anxiety measured, was the alpha bigger than 0.60?

- No (0): the alpha reported for these measures was below 0.60
- Yes (1): the measures obtained have an alpha of 0.60 or higher

Domain 5 – Selection of the reported results

1. Are there missing analyses in the results section, which have been mentioned in the introduction/methods section (check hypotheses, data analyses or preregistration)? (when you assess with x is related to a b and c but only report association x to a)

- No (0): there are some analyses which have been left out in the results section, although they have been planned and named beforehand
- Yes (1): all measurements planned (mentioned in the introduction/methods section) are also reported in the results

1. Did they refrain from applying more analyses which have not been planned beforehand (check hypotheses, data analyses or preregistration) (when you assess whether x is related to a but report x is related to a b and c)

- No (0): here is evidence that measurements were analyzed in multiple ways despite not planning them apriori (no mention of them in intro and method). In the result section these extra analyses are not described as post-hoc but as main results.
- Yes (1): all analyses planned match all the data

Calculation of Quality score:

- Add all points for yes together and weigh score (since domain 2.2 and domain 4.1 are only applicable for specific studies/situations).

Table S1. Quality Assessment of Individual Studies

| Background | Randomization | | Deviations | | Dropout | Outcome Measurement | | Reported Results | | Quality |
| --- | --- | --- | --- | --- | --- | --- | --- | --- | --- | --- |
| Author | 1.a | 1.b | 2.a | 2.b | 3.a | 4.a | 4.b | 5.a | 5.b |  |
| Aktar et al. (2013) | 1/1 | NA | 1/1 | NA | 1/1 | 1/1 | 2/2 | 1/1 | 1/1 | 100% |
| Aktar et al. (2014) | 1/1 | NA | 1/1 | NA | 1/1 | 1/1 | 2/2 | 1/1 | 1/1 | 100% |
| Aktar et al. (2018) | 1/1 | NA | 1/1 | NA | 1/1 | 1/1 | 2/2 | 1/1 | 1/1 | 100% |
| Blackford & Walden (1998) | 1/1 | NA | 1/1 | NA | 1/1 | 1/1 | 1/1 | 1/1 | 1/1 | 100% |
| Carpenter (2004) | 1/1 | NA | 1/1 | 1/1 | 1/1 | 1/1 | 1/1 | 1/1 | 1/1 | 100% |
| De Rosnay et al. (2006) | 1/1 | NA | 1/1 | 1/1 | 1/1 | 1/1 | 1/1 | 1/1 | 1/1 | 100% |
| Dubi et al. (2008) | 1/1 | NA | 1/1 | NA | 1/1 | 1/1 | 2/2 | 1/1 | 1/1 | 100% |
| Gerull & Rapee (2002) | 1/1 | NA | 1/1 | NA | 1/1 | 1/1 | 1/1 | 1/1 | 1/1 | 100% |
| Goodman-Wilson (2012) | NA | 1/1 | 1/1 | 1/1 | 1/1 | 1/1 | 2/2 | 1/1 | 1/1 | 100% |
| Hirshberg & Svedja (1990) | 1/1 | NA | 0/1 | NA | 1/1 | 1/1 | NA | 1/1 | 1/1 | 83% |
| Kim et al. (2010) | NA | 1/1 | 0/1 | NA | 0/1 | 1/1 | NA | 1/1 | 1/1 | 67% |
| Klinnert (1984) | 1/1 | NA | 0/1 | NA | 1/1 | 1/1 | NA | 1/1 | 0/1 | 67% |
| Knieps et al. (1994) | 1/1 | NA | 1/1 | NA | 1/1 | 1/1 | NA | 1/1 | 1/1 | 100% |
| Möller et al. (2014) | 1/1 | NA | 1/1 | NA | 1/1 | 1/1 | 2/2 | 1/1 | 1/1 | 100% |
| Mumme et al. (1996) | NA | 1/1 | 1/1 | NA | 1/1 | 1/1 | NA | 1/1 | 0/1 | 83% |
| Murray et al. (2008) | 1/1 | NA | NA | 1/1 | 1/1 | 1/1 | 2/2 | 1/1 | 1/1 | 100% |
| Rosen et al. (1992) | 1/1 | NA | 1/1 | NA | 1/1 | 1/1 | NA | 1/1 | 0/1 | 83% |
| Sorce et al. (1985) | NA | 1/1 | 1/1 | NA | 0/1 | 1/1 | NA | 1/1 | 0/1 | 67% |
| Stenberg (2003) | NA | 1/1 | 1/1 | NA | 1/1 | 1/1 | 1/1 | 1/1 | 1/1 | 100% |
| Walden & Ogan (1988) | 1/1 | NA | 1/1 | NA | 1/1 | 1/1 | NA | 1/1 | 1/1 | 100% |
| Walden & Baxter (1989) | NA | 1/1 | 1/1 | NA | 1/1 | 1/1 | NA | 1/1 | 1/1 | 100% |
| Walden et al. (1991) | 1/1 | NA | 1/1 | NA | 1/1 | 1/1 | NA | 1/1 | 1/1 | 100% |
| Zarbatany & Lamb (1985) | NA | 1/1 | 1/1 | NA | 0/1 | 1/1 | NA | 1/1 | 1/1 | 83% |

*Note*: NA = Not applicable.

**C**

Figure C1. Funnel and forest plots of main effects on child fear in experimental studies


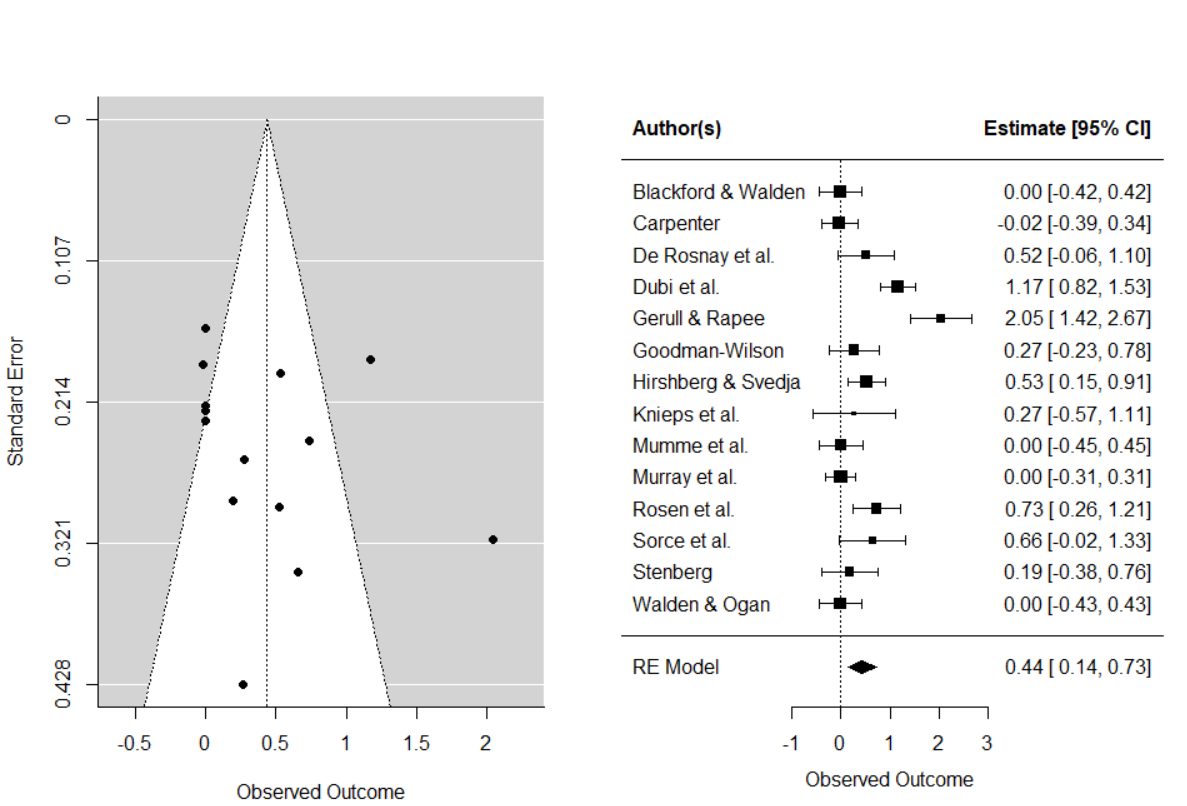


Figure C2. Funnel and forest plots of BI effect on child fear in experimental studies


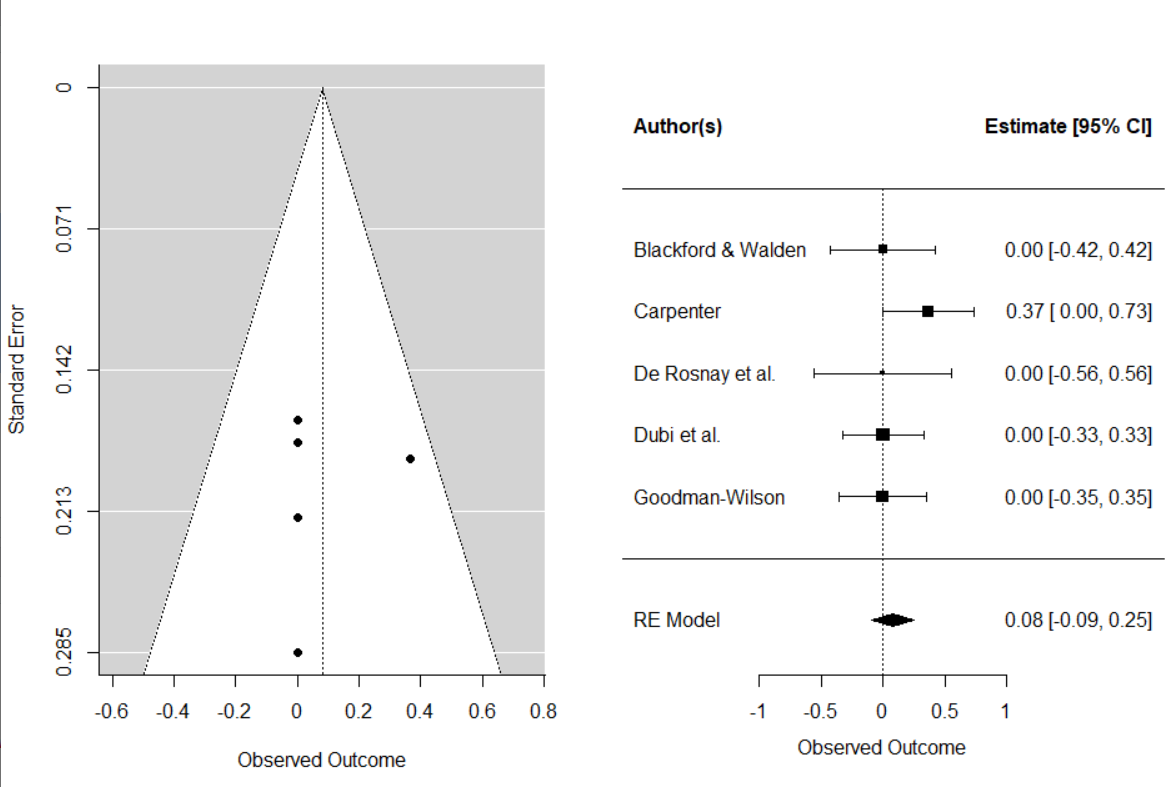


Figure C3. Funnel and forest plots of main effects on child avoidance in experimental studies


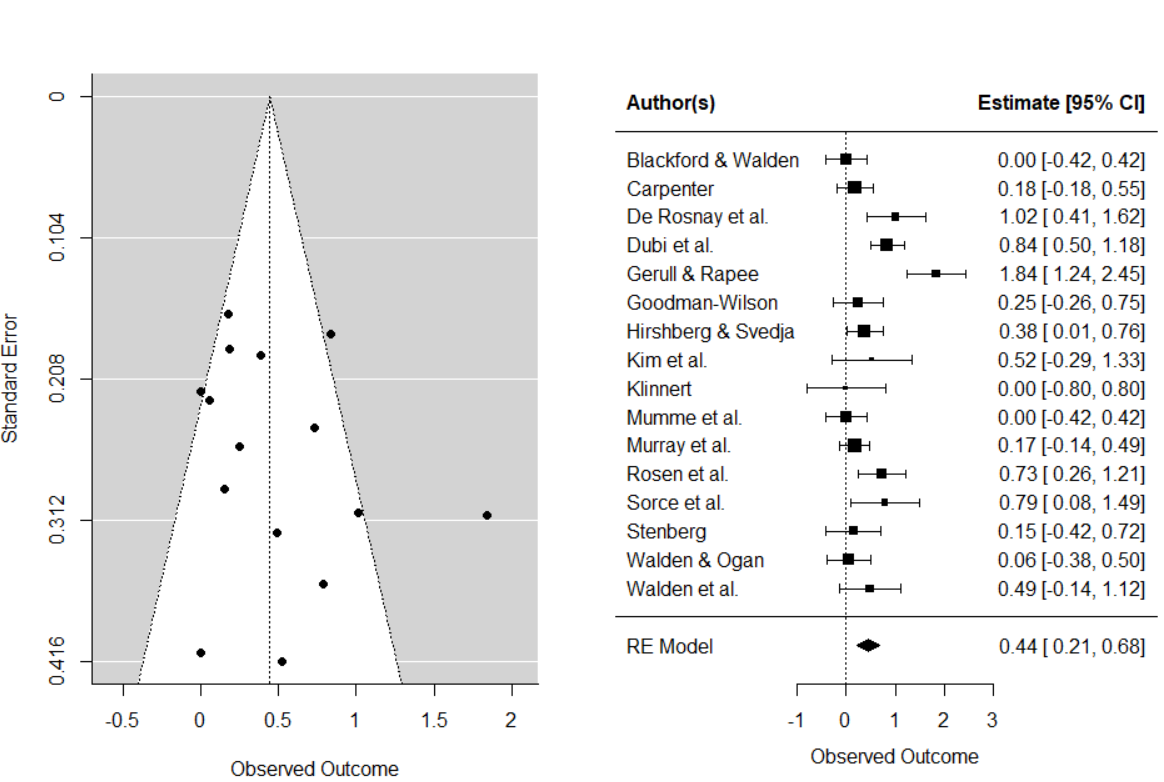


Figure C4. Funnel and forest plots of BI effect on child avoidance in experimental studies


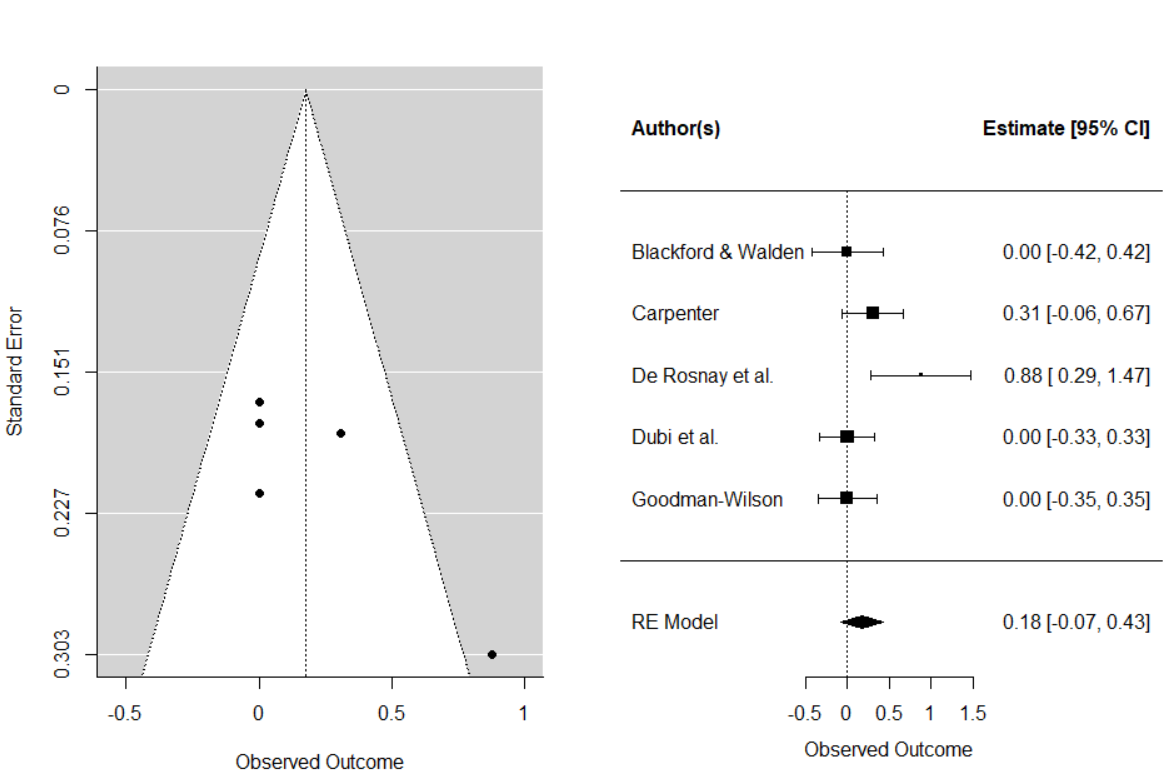

Supplement: Supplementary file 1 — Supplementary file1 (DOCX 672 KB) [file 10567_2023_448_MOESM1_ESM.docx]
